# 5-Aminolevulinic Acid Fluorescence in High Grade Glioma Surgery: Surgical Outcome, Intraoperative Findings, and Fluorescence Patterns

**DOI:** 10.1155/2014/232561

**Published:** 2014-04-08

**Authors:** Alessandro Della Puppa, Pietro Ciccarino, Giuseppe Lombardi, Giuseppe Rolma, Diego Cecchin, Marta Rossetto

**Affiliations:** ^1^Department of Neurosurgery, Padua University Hospital, Azienda Ospedaliera di Padova, Via Giustiniani 2, 35128 Padova, Italy; ^2^Department of Oncology, IOV IRCCS Oncology Institute of Padua, Via Gattamelata 64, 35128 Padova, Italy; ^3^Neuroradiology Unit, Padua University Hospital, Via Giustiniani 2, Padua, 35128 Padova, Italy; ^4^Department of Medicine (DIMED), Nuclear Medicine Service, University of Padua, Via Giustiniani 2, 35128 Padova, Italy

## Abstract

*Background*. 5-Aminolevulinic acid (5-ALA) fluorescence is a validated technique for resection of high grade gliomas (HGG); the aim of this study was to evaluate the surgical outcome and the intraoperative findings in a consecutive series of patients. *Methods*. Clinical and surgical data from patients affected by HGG who underwent surgery guided by 5-ALA fluorescence at our Department between June 2011 and February 2014 were retrospectively evaluated. Surgical outcome was evaluated by assessing the resection rate as gross total resection (GTR) > 98% and GTR > 90%. We finally stratified data for recurrent surgery, tumor location, tumor size, and tumor grade (IV versus III grade sec. WHO). *Results*. 94 patients were finally enrolled. Overall GTR > 98% and GTR > 90% was achieved in 93% and 100% of patients. Extent of resection (GTR > 98%) was dependent on tumor location, tumor grade (*P* < 0.05), and tumor size (*P* < 0.05). In 43% of patients the boundaries of fluorescent tissue exceeded those of tumoral tissue detected by neuronavigation, more frequently in larger (57%) (*P* < 0.01) and recurrent (60%) tumors. *Conclusions*. 5-ALA fluorescence in HGG surgery enables a GTR in 100% of cases even if selection of patients remains a main bias. Recurrent surgery, and location, size, and tumor grade can predict both the surgical outcome and the intraoperative findings.

## 1. Introduction

High grade gliomas are extremely aggressive lesions and represent the most common primary malignant brain tumors with an annual incidence of 5.26/100.000/year. Despite several efforts on surgical techniques, oncological therapies, and molecular understandings prognosis is poor. Among factors affecting patients' outcome, extent of surgical removal and functional preservation have been proved to have a great impact; therefore, continuous refinements of surgical approaches and techniques are investigated [1–3].

The extent of tumor resection (EOR) is proved to be an important prognostic factor in HGG. Therefore, several neurosurgeons tried to quantify the EOR through a threshold of resection correlated with a significant improvement in overall survival. One of the most comprehensive series set this threshold at 95–100% rate of resection [4] with a median survival of 13 months compared with 8.8 months in patients with a EOR < 98%. However, Sanai and colleagues clearly demonstrated that in selected newly diagnosed HGG patient OS begins to benefit from an EOR ≥ 78% and that this trend of improvement increases at highest levels of resection [5].

The role of surgery on recurrent HGG is still debated. However, in recurrent GBMs, some authors showed a more favorable response to chemotherapy in patients harboring a residual disease <10 cm^3^ [6]. Moreover, a great association between survival and recurrence with EOR of 70% and residual volume (RV) of 5 cm^3^ is proved to occur as recently demonstrated in a large series of Johns Hopkins' group [7].

5-Aminolevulinic acid (5-ALA) is a natural precursor of haemoglobin that produces synthesis and accumulation of fluorescent porphyrins, particularly PPIX. The concentration of PPIX is elevated in several tumoral tissues such as HGG, and in central nervous system its concentration is significantly higher in malignant tissue than in normal brain. PPIX fluorescence can be visualized through a modified surgical microscope able to shift from a white light to a violet-blue illumination using a dielectric 440 nm short-pass filter into the illumination path. Intraoperatively, under white light, no fluorescence is visible while shifting at violet-blue illumination tumor tissue fluorescing and three main patterns can be distinguished: necrotic area that usually displays no or only inhomogeneous red fluorescence, solid viable tumor showing bright fluorescence intensity, and the transitional area of infiltrating and invasive brain tissue appearing as “vague” fluorescence. From a pathological point of view, these three different fluorescence patterns have been studied and correlated with tissue specimens. In solid tumor and invasive areas, 5-ALA showed a positive predictive value of 100% and 97%, respectively, while in normal tissue a negative predictive value of 67% [8] was found.

In 2006, Stummer and colleagues published the first phase III trial on resection and survival in 5-ALA-assisted glioma surgery. Their randomized controlled multicentric study evidenced that a total removal of the tumor was achieved in 65% of patients assigned 5-ALA compared with 36% in patients who underwent traditional surgery [9]. Actually, level 2b evidence shows that 5-ALA-assisted surgery is more effective than conventional surgery in increasing EOR and prolonging overall survival in GBM [10].

## 2. Materials and Methods

### 2.1. Patient Population

We screened for surgery with 5-ALA all patients consulting our unit whose MRI scans were suggestive of HGG, from June 2011 to February 2014. Resectability was decided over T1-weighted gadolinium Magnetic Resonance Imaging (T1-GdMRI). Inclusion criteria for enrolment were MRI suggestive for high grade glioma (HGG) both newly diagnosed and recurrent ones, gross total surgical removal (GTR) (i.e., >90%) deemed possible at preoperative assessment, and plan for surgery guided by 5-ALA fluorescence with the assistance of MRI neuronavigation (T1Gd). Tumor location was analyzed on contrast-enhanced T1 sequences and the proximity to eloquent structures was assessed with diffusion tensor and functional Magnetic Resonance Imaging (MRI) images. According to previous authors [4, 13, 14] were regarded as eloquent areas: primary motor and sensory cortex, the basal ganglia, thalamus, hypothalamus, cerebral peduncles, the brainstem, the dentate nucleus, the presumed language areas (identified by fMRI), the primary visual cortex, and essential white matter tracts linked to these eloquent regions (identified by DTI). Patients were divided into “patients with tumor in eloquent areas” when tumor was into or close (less than 10 mm) to eloquent structures and “patients with tumor in noneloquent areas” when tumor was more far than 10 mm from eloquent areas. Tumor location and extent of resection were defined by an expert neuroradiologist based on pre- and postoperative Magnetic Resonance Imaging (MRI). Informed consent was obtained from all patients. Tumor grade was histologically confirmed in all cases.

### 2.2. Surgical Strategy

Patients were administered orally 20 mg/kg 5-aminolevulinic acid 2–4 h before surgery, as previously described [15]. All operations were performed with a Zeiss Pentero microscope equipped with a fluorescent 400 nm UV light and filters. All patients were operated on in a MRI neuronavigational setting. Diffusion tensor and functional Magnetic Resonance Imaging (MRI) were performed to visualize functional cortical areas and cerebral fiber tracts, and subsequently loaded in the neuronavigational system according to tumor location. Microsurgical removal was started using standard white xenon light and switched to the violet-blue excitation light whenever tumor boundaries were visually indistinct from healthy brain tissue. At the end of resection, the cavity was systematically checked in the violet-blue light mode for any residual tumor. In the cases in which resection was stopped and residual fluorescent tissue was still present in the boundaries, the fluorescence intensity was recorded (as bright or vague fluorescence) [15]. In all cases, at the end of resection neuronavigational record of surgical boundaries was carried out. With regard to the functional area involved, intraoperative monitoring was performed either in asleep or in awake surgery, depending on both patient and tumor features. A standard intraoperative surgical and neurophysiological protocol was followed for patients with tumor in eloquent areas. Such protocol entailed MRI neuronavigation and continuous electroencephalography (EEG), electrocorticography (ECoG), and multichannel electromyography recordings. Monitoring included tracking of Motor Evoked Potentials (MEPs), sensory evoked potentials (SEPs), and cortical and subcortical stimulation as previously reported [16]. Two criteria were followed to stop resection: first, the lack of tumoral tissue at white light and of fluorescent tissue at final blue light control. The second one was the localization of either a functional area or a cortical tract during fluorescent tissue resection. Pre- and postoperative MRI formal reports were reevaluated by a neuroradiologist. In order to compare 5-ALA fluorescence and neuronavigation data with regard to identification of surgical boundaries, pre- and postoperative GdT1 MRI images, and intraoperative neuronavigation data at the end of resection, were finally compared by an expert neuroradiologist.

### 2.3. Research Variables

Our study focused on defining the extent of resection and intraoperative fluorescence patterns in patients affected by HGG who underwent surgery guided by 5-ALA fluorescence and assisted by MRI neuronavigation and neurophysiological monitoring. The assessment of the extent of resection was carried out by MRI studies with a 1.5 T GE scanner. According to previous authors [4, 17, 18] extent of resection was reported as gross total resection more than 98% (GTR > 98%), and gross total Resection more than 90% (GTR > 90%). Subtotal resection was a resection less than 90% with a residual volume more than 10%. All MRI studies included Fluid Attenuated Inversion Recovery, T2-weighted and T1-weighted, before and after administration of gadolinium (Gd) contrast medium (gadopentetate dimeglumine). A volumetric Gd T1-weighted MRI study was finally performed. Control MRI was performed within 72 h from surgery, in order to evaluate degree of removal. The extent of resection of enhancing tissue was carefully measured by an expert neuroradiologist, comparing volumetric postoperative magnetic resonance imaging (MRI) with volumetric preoperative MRI.

Intraoperative fluorescence data were registered and retrospectively evaluated on video records. In particular, the different fluorescent nuances and the pattern of fluorescence were assessed according to previous reports [8, 19].

The extent of resection of enhancing tissue was carefully measured by an expert neuroradiologist, comparing volumetric postoperative magnetic resonance imaging (MRI) with volumetric preoperative MRI.

In order to compare 5-ALA fluorescence and neuronavigation data with regard to identification of surgical boundaries, pre- and postoperative GdT1 MRI images and intraoperative neuronavigation data at the end of resection were finally compared by an expert neuroradiologist.

Our study finally stratified the data for specific subgroups of patients. With this purpose, recurrent surgery, tumor location (eloquent versus noneloquent), tumor size (< versus > or = 9 cm^3^), and tumor grade (IV versus III WHO grade) [20] were analyzed. We calculated the median tumoral volume through a 3D volumetric measurement of preoperative MR imaging studies by the modified ellipsoid volume equation as follows: (*A* × *B* × *C*)/2, where *A*, *B*, and *C* represent the 3 largest orthogonal diameters of the lesion.

### 2.4. Statistical Analysis

Statistical analysis to compare the categorical variables was performed via *χ*² test in two-way tables. If any value was <10 Fisher's exact test was used (2-sided). A *P* value <0.05 was considered significant.

## 3. Results

Ninety-four patients (53 males and 41 females) were enrolled (Table 1). The median age was 58 years (range 27–79 years). 61 patients were affected by newly diagnosed gliomas, while 33 patients had second surgery for recurrent glioma.

Histopathological results showed 81 patients harbored glioblastomas (WHO Grade IV) and 13 grade III gliomas (astrocytoma, oligoastrocytoma, and oligodendroglioma WHO Grade III). 43 patients presented a tumor growing in an “eloquent area” whilst 51 patients did not. Surgery was assisted by neurophysiological monitoring in all cases of eloquent area tumors (43/43) and in 45/51 (88%) of noneloquent area tumors.

### 3.1. Extent of Resection

Extent of resection data is summarized in Table 2. Patients were divided into two groups according to extent of resection. Gross total removal > 90% was achieved in all (94/94) patients enrolled in the study. Gross total removal >98% was achieved in 88 patients (93%).

**Figure 1:**
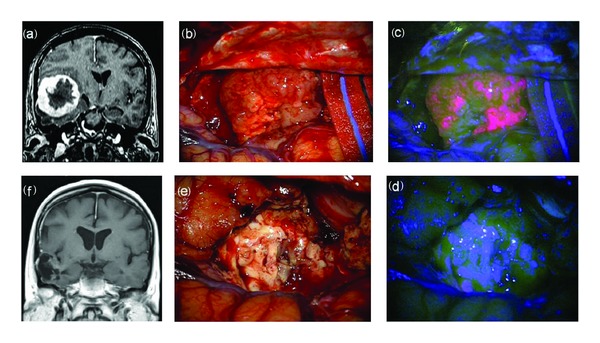
F 63/Y, preoperative MRI scan with gadolinium showing a large temporal high grade glioma (a). Intraoperative view: tumor resection under white (b) and blue (c) light till the complete removal of lesion. Final view at blue (d) and white light (e). Postoperative MRI scan with gadolinium (f) showing the complete resection of tumor. Histological report: glioblastoma (astrocytoma grade IV sec WHO).

In particular, GTR > 98% was achieved in 39/43 (90%) of patients with tumor located in eloquent areas and in 49/51 (96%) of patients with tumor located in noneloquent areas (*P* = 0.407) (Figure 1), in 57/61 (93%) and in 31/33 (94%) of patients affected by newly diagnosed and recurrent glioma, in 78/81 (96%) and in 10/13 (79%) of patients affected by a grade IV and grade III glioma (*P* = 0.018), in 45/51 (88%) and in 43/43 (100%) of patients affected by a larger (≥9 cm^3^) and smaller (<9 cm^3^) tumor, respectively (*P* = 0.029).

**Figure 2:**
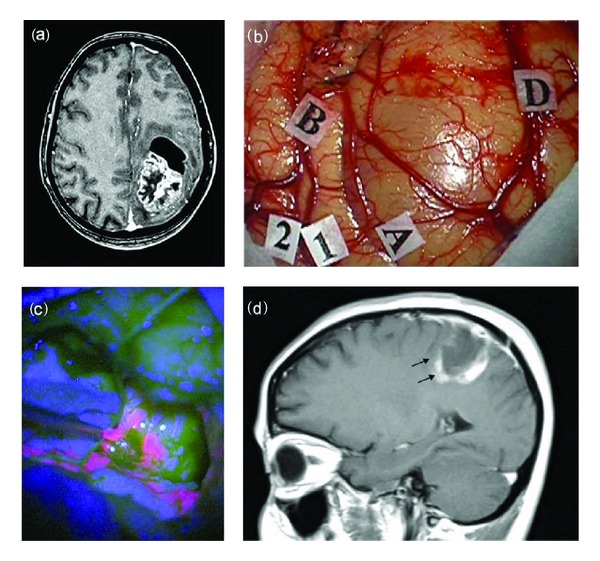
F 47/Y, preoperative MRI scan with gadolinium showing a large frontoparietal glioma (a). Intraoperative view: (b) identification by MRI-navigation of tumor boundaries on the cortical surface (markers A; B; D) and identification by mapping of surrounding motor area (1, 2). Resection under blue-light (c) showing a bright fluorescent tumor. The postoperative MRI scan (d) shows a residual tumor (black arrows). Histological report: glioblastoma (astrocytoma grade IV sec WHO).

As far as tumors located in eloquent areas are concerned, in 39 out of 43 patients the resection was stopped because a functional area or cortical tract was identified or because MEP amplitudes were reduced in an area where fluorescent tumor cells were still visible (Figure 2). In all these patients, bright fluorescent tissue was detected at final control.

### 3.2. Fluorescent Data

All tumours presented fluorescent under blue light. Bright fluorescence was detected in all patients, whilst vague fluorescence was reported in only 81/94 (86%) of cases.

The 3-layer model of fluorescence was reported in 63% of cases (60/94): in 54/61 (88%) and in 6/33 (18%) of patients affected by newly diagnosed and recurrent glioma (*P* < 0.001), in 60/81 (74%) and in 0/13 (0%) of patients affected by a grade IV and grade III glioma (*P* < 0.001), and in 32/51 (62%) and in 28/43 (65%) of patients affected by a larger (≥9 cm^3^) and smaller (<9 cm^3^) tumor, respectively.

### 3.3. Tumour Boundaries

**Figure 3:**
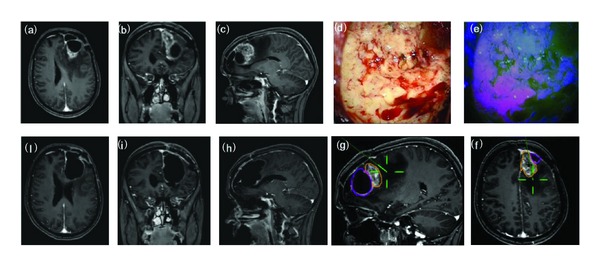
F/43Y, preoperative MRI scan with gadolinium showing a recurrent frontal glioblastoma (a–c). Intraoperative view: tumor resection under white (d) and blue (e) light. Intraoperative boundaries of surgical cavity at MRI navigation after resection under blue light (f-g). Postoperative MRI scan with gadolinium (h–j) showing the complete tumor resection and the postsurgical cavity roughly larger than tumor size at preoperative imaging. Histological report: recurrent glioblastoma (astrocytoma grade IV sec WHO).

The tumor boundaries detected by neuronavigation differed by fluorescence data in 43% of cases (41/94). In all 41 patients fluorescent tissue was detected over boundaries identified by neuronavigation. Conversely, neuronavigation never showed tumor tissue in not fluorescent areas. The divergence between neuronavigation and 5-ALA fluorescence was reported in 21/61 (34%) and in 20/33 (60%) of patients affected by newly diagnosed and recurrent glioma (*P* = 0.845), in 34/81 (42%) and in 7/13 (53%) of patients affected by a grade IV and grade III glioma (*P* = 0.549), and in 29/51 (57%) and in 12/43 (28%) of patients affected by a larger (>9 cm^3^) and smaller (<9 cm^3^) tumor, respectively (*P* < 0.01) (Figure 3).

## 4. Discussion

### 4.1. Our Present Relevant Results

GTR > 90% was achieved in 100% of patients whilst GTR > 98% was achieved in 93% of cases. Even if GTR > 98% was greater between patients affected by tumors in noneloquent areas (96 versus 90%), the difference was not statistically significant. Based on our results extent of resection rate was significantly higher in IV grade gliomas (*P* = 0.029) and in smaller tumors (*P* = 0.018). Concerning divergence between neuronavigation and 5-ALA fluorescence, we found a significant difference just regarding tumor volume, in particular in lesion >9 cm^3^ (*P* < 0.01) while WHO grade and recurrent surgery were not statistically significant. Finally, the 3-layer pattern of fluorescence was more frequently preserved in newly diagnosed tumors compared to recurrence (*P* < 0.001) and in IV grade than in III grade gliomas (*P* < 0.001).

### 4.2. Comparison of Our Relevant Results to Those of Other Studies

GTR > 98% rate in our series was achieved in 93% of cases considering patients harboring lesions in both eloquent and noneloquent areas. These data have been found by several other works that focused on the utility of 5-ALA in gliomas surgery [12, 13]. However, as shown in Table 2, resection rate data are strictly dependent on the main bias of the proportion of patients with HGG in eloquent and noneloquent area in each study. In fact, as accurately reported by Schucht and coworkers [13], overall resection rate (contrast resection enhanced tumor—CRET) in their population was 89%: in noneloquent tumors CRET rate was 97% while it clearly decreased up to 74% in eloquent areas gliomas. These data however should be read according to the largeness of each study. In fact Stummer and colleagues reported a 65% of GTR, but it should be considered that this GTR rate arises from the largest and unique phase III trial and it reflects the surgical achievements of several Institutions with different expertise [9]. On the other hand, it seems that the increasing rate of GTR reported by these studies may arise from a growing experience in modern techniques of intraoperative mapping/monitoring and fluorescence-guided surgery. As a matter of fact, functional preservation and maintaining quality of life are undoubtedly a mandatory goal in glioma surgery since patients harboring HGG have a limited life expectancy. However, function protection can limit EOR in critical area gliomas. Stummer and colleagues found that, in 5-ALA assisted surgery, residual tumor volumes on postoperative MRI were greater in patients with glioma in eloquent regions compared with patients harboring tumor in noneloquent regions. In their study proximity to eloquent areas was the only independent factor on residual tumor [15]. Therefore, surgery assisted by both 5-ALA and neurophysiological monitoring represents an increasing interested field. In 2010 Feigl and coworkers performed this multimodal approach (5-ALA associated with motor evoked potentials and cortical and subcortical stimulation) in 18 patients with HGG in critical areas obtaining a GTR in 64% of cases while in 24% of cases surgical removal was stopped according to neurophysiological data and impairment was recorded in about 11% of cases [11]. Recently, we published our series of 31 patients with HGG in eloquent areas treated with this multimodal approach. We achieved a GTR (>98%) in 93% of cases and we stopped surgery in 26% to avoid neurological deficits. Finally, a postoperative impairment was found in 3% at 3-month follow-up [16]. Other authors, even if considering gliomas both in eloquent and in noneloquent areas, focused on 5-ALA and neurophysiological assisted surgery providing satisfactory and safe results [12, 13] compared to other multimodal approaches such as intraoperative MRI and neurophysiological monitoring [21].

### 4.3. MR Contrast Enhanced Tumor and 5-ALA Fluorescence

Analyzing our results we found a significant divergence between 5-ALA fluorescence and MR contrast enhanced tumor according to neuronavigation data. In particular considering lesions >9 cm^3^ we discovered that 5-ALA fluorescing tissue expanded significantly over tumor volume scheduled by contrast enhanced tumor at MRI (*P* = 0.01) (Figure 3). As well known [22] during surgery volumetric deformations occur and brain morphology changes through a dynamic process called brain shift. This phenomenon determines a continuous modification of cerebral structures and therefore a progressive inaccuracy of neuronavigation data resulting in no longer trusted during surgical intervention. This finding has a particular relevance since neuronavigation data are based on uploaded contrast enhanced tumor images. As reported by a recent study focused on this topic 5-ALA-guided surgery extends up to 6 mm beyond the MRI contrast enhanced tumor [23]. A more extended tumor resection undoubtedly risks function preservation especially in critical areas. Therefore, neurophysiological monitoring represents a synergistic tool maximizing resection and minimizing neurological deficits.

### 4.4. Indications for Future Research

**Figure 4:**
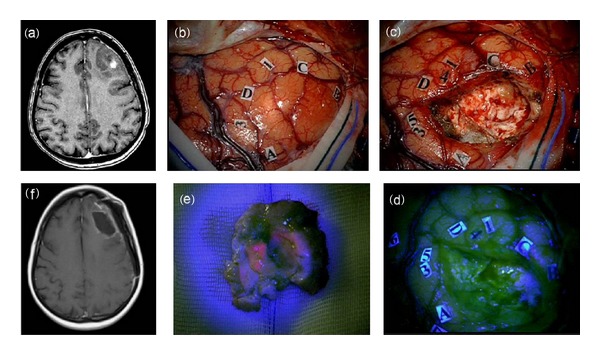
F 47/Y, preoperative MRI scan with gadolinium showing a left frontal high grade glioma (a). Intraoperative view: (b) identification by MRI-navigation of tumor boundaries on the cortical surface (markers A, B, C, D) and identification by mapping in awake surgery of surrounding language areas (1–5). (c) Final inspection of the surgical cavity under blue-light (d) showing no fluorescent residual tumor. The tumor at blue-light inspection showed a fluorescent inner part corresponding to enhancing component at preoperative MRI (a). The postoperative MRI scan (f) confirms the complete resection of tumor (f). Histological report: anaplastic astrocytoma (astrocytoma grade III sec WHO).

In our experience we found a definitely more constant 3-layer pattern of fluorescence in newly diagnosed IV grade than in III grade (Figure 4) and recurrent gliomas, as a peculiar and characteristic pattern of fluorescence of these tumors. According to previous studies on GBM in fact, focusing on pathological morphology and spatial distribution in tumor bulk, a three-layer concentric model was found due to a different microenvironmental regulation [24]. Furthermore, we found a resection rate of grade III lower than grade IV gliomas. Literature lacks studies focusing on Grade III gliomas resection guided by 5-ALA fluorescence and our study was not designed to do that. However, recent findings documented a possible use of 5-ALA in detecting anaplastic foci within LGG with no evidence of preoperative contrast enhancement [25]. Widhalm and colleagues studied 17 patients with grad II and III gliomas without focal contrast enhancement and they found that focal 5-ALA fluorescence was a significant factor differentiating grade 2 and grade 3 gliomas. A main issue and surgical trick emerging by our data was the lower rate of resection in larger tumors. Probably, the collapse of the surgical cave during resection does not allow maintaining the blue light perpendicular illumination of tumor representing the ideal situation for identification of pathological tissue.

## 5. Conclusions

5-ALA fluorescence in HGG surgery can achieve a GTR in 100% of cases even if selection of patients remains a main bias. Indeed, only patients in whom gross total surgical removal was deemed possible at preoperative assessment were enrolled in the study. Recurrent surgery, tumor location, tumor size, and tumor grade are variables that in our experience can predict both the surgical outcome and the intraoperative findings. Our data must be confirmed by further studies.

## Figures and Tables

**Table 1:** Clinical and surgical characteristics of patients.

Parameter	No. (%)
Number of patients	94
Male/female	53/41
Mean age	58
Newly diagnosed HGG	61
Recurrent HGG	33
Tumor grade	
GBM	81
Grade III glioma	13
Astrocytoma	7
Oligoastrocytoma	3
Oligodendroglioma	3
Tumor location	
Eloquent area	43
Noneloquent area	51
Monitoring assisted surgery	
Eloquent area tumors	43 (100%)
Noneloquent area tumors	45 (88%)

**Table 2:** Resection rates reported in recent publications basing on tumors localization.

Authors, year	Rate of resection		
	All cerebral localizations	Noneloquent areas	Eloquent areas
Stummer et al., 2006 [9]	65%°	69%°	61%°
Feigl et al., 2010 [11]	—	—	64%*
Díez Valle et al., 2011 [12]	100%*	—	—
Schucht et al., 2012 [13]	89%°	97%°	74%°
Present series	93%*	96%*	90%*

°Contrast resection enhanced tumor (CRET); *gross total removal >98%; —: not reported.

## References

[1] Sanai N, Berger MS (2008). Glioma extent of resection and its impact on patient outcome. *Neurosurgery*.

[2] Hardesty DA, Sanai N (2012). The value of glioma extent of resection in the modern neurosurgical era. *Frontiers in Neurology*.

[3] McGirt MJ, Mukherjee D, Chaichana KL, Than KD, Weingart JD, Quinones-Hinojosa A (2009). Association of surgically acquired motor and language deficits on overall survival after resection of glioblastoma multiforme. *Neurosurgery*.

[4] Lacroix M, Abi-Said D, Fourney DR (2001). A multivariate analysis of 416 patients with glioblastoma multiforme: prognosis, extent of resection, and survival. *Journal of Neurosurgery*.

[5] Sanai N, Polley M, McDermott MW, Parsa AT, Berger MS (2011). An extent of resection threshold for newly diagnosed glioblastomas: clinical article. *Journal of Neurosurgery*.

[6] Keles GE, Lamborn KR, Chang SM, Prados MD, Berger MS (2004). Volume of residual disease as a predictor of outcome in adult patients with recurrent supratentorial glioblastomas multiforme who are undergoing chemotherapy. *Journal of Neurosurgery*.

[7] Chaichana KL, Jusue-Torres I, Navarro-Ramirez R (2014). Establishing percent resection and residual volume thresholds affecting survival and recurrence for patients with newly diagnosed intracranial glioblastoma. *Neuro-Oncology*.

[8] Idoate MA, Díez Valle R, Echeveste J, Tejada S (2011). Pathological characterization of the glioblastoma border as shown during surgery using 5-aminolevulinic acid-induced fluorescence. *Neuropathology*.

[9] Stummer W, Pichlmeier U, Meinel T, Wiestler OD, Zanella F, Reulen H (2006). Fluorescence-guided surgery with 5-aminolevulinic acid for resection of malignant glioma: a randomised controlled multicentre phase III trial. *The Lancet Oncology*.

[10] Stummer W, Reulen H, Meinel T (2008). Extent of resection and survival in glioblastoma multiforme: identification of and adjustment for bias. *Neurosurgery*.

[11] Feigl GC, Ritz R, Moraes M (2010). Resection of malignant brain tumors in eloquent cortical areas: a new multimodal approach combining 5-aminolevulinic acid and intraoperative monitoring. *Journal of Neurosurgery*.

[12] Díez Valle R, Tejada Solis S, Idoate Gastearena MA, García de Eulate R, Domínguez Echávarri P, Aristu Mendiroz J (2011). Surgery guided by 5-aminolevulinic fluorescence in glioblastoma: volumetric analysis of extent of resection in single-center experience. *Journal of Neuro-Oncology*.

[13] Schucht P, Beck J, Abu-Isa J (2012). Gross total resection rates in contemporary glioblastoma surgery: results of an institutional protocol combining 5-ALA intraoperative fluorescence imaging and brain mapping. *Neurosurgery*.

[14] Sawaya R, Hammoud M, Schoppa D (1998). Neurosurgical outcomes in a modern series of 400 craniotomies for treatment of parenchymal tumors. *Neurosurgery*.

[15] Stummer W, Novotny A, Stepp H, Goetz C, Bise K, Reulen HJ (2000). Fluorescence-guided resection of glioblastoma multiforme by using 5-aminolevulinic acid-induced porphyrins: a prospective study in 52 consecutive patients. *Journal of Neurosurgery*.

[16] della Puppa A, de Pellegrin S, d'Avella E (2013). 5-aminolevulinic acid (5-ALA) fluorescence guided surgery of high-grade gliomas in eloquent areas assisted by functional mapping. Our experience and review of the literature. *Acta Neurochirurgica*.

[17] Butowski N, Lamborn KR, Berger MS, Prados MD, Chang SM (2007). Historical controls for phase II surgically based trials requiring gross total resection of glioblastoma multiforme. *Journal of Neuro-Oncology*.

[18] Vogelbaum MA, Jost S, Aghi MK (2012). Application of novel response/progression measures for surgically delivered therapies for gliomas: Response Assessment in Neuro-Oncology (RANO) working group. *Neurosurgery*.

[19] Rampazzo E, della Puppa A, Frasson C (2014). Phenotypic and functional characterization of Glioblastoma cancer stem cells identified through 5-aminolevulinic acid-assisted surgery. *Journal of Neuro-Oncology*.

[20] Louis DN, Ohgaki H, Wiestler OD (2007). The 2007 WHO classification of tumours of the central nervous system. *Acta Neuropathologica*.

[21] Senft C, Forster M, Bink A (2012). Optimizing the extent of resection in eloquently located gliomas by combining intraoperative MRI guidance with intraoperative neurophysiological monitoring. *Journal of Neuro-Oncology*.

[22] Nabavi A, Black PM, Gering DT (2001). Serial intraoperative magnetic resonance imaging of brain shift. *Neurosurgery*.

[23] Schucht P, Knittel S, Slotboom J (2014). 5-ALA complete resections go beyond MR contrast enhancement: shift corrected volumetric analysis of the extent of resection in surgery for glioblastoma. *Acta Neurochirurgica*.

[24] Persano L, Rampazzo E, della Puppa A, Pistollato F, Basso G (2011). The three-layer concentric model of glioblastoma: cancer stem cells, microenvironmental regulation, and therapeutic implications. *TheScientificWorldJournal*.

[25] Widhalm G, Wolfsberger S, Minchev G (2010). 5-aminolevulinic acid is a promising marker for detection of anaplastic foci in diffusely infiltrating gliomas with nonsignificant contrast enhancement. *Cancer*.

